# Vasorelaxant and Hypotensive Mechanisms of *Nelumbo nucifera* Seed Extract: Roles of Nitric Oxide, Calcium Channel Blockade and eNOS Interaction with Active Compounds

**DOI:** 10.3390/ph18101500

**Published:** 2025-10-06

**Authors:** Usana Chatturong, Nitra Nuengchamnong, Anjaree Inchan, Kittiwoot To-On, Tippaporn Bualeong, Wiriyaporn Sumsakul, Anyapat Atipimonpat, Kittiphum Meekarn, Yasuteru Shigeta, Kowit Hengphasatporn, Sarawut Kumphune, Krongkarn Chootip

**Affiliations:** 1 Department of Physiology, Faculty of Medical Science and Center of Excellence for Innovation in Chemistry, Naresuan University, Phitsanulok 65000, Thailand; chatturong.u@gmail.com (U.C.); kittiwoott62@nu.ac.th (K.T.-O.); tippapornb@gmail.com (T.B.); 2 Science Laboratory Centre, Faculty of Science, Naresuan University, Phitsanulok 65000, Thailand; nitran@nu.ac.th; 3 Faculty of Medicine, Ministry of Public Health, Praboromarajchanok Institute, Nonthaburi 11000, Thailand; anjaree.inc@pi.ac.th; 4 Expert Center of Innovative Herbal Products, Thailand Institute of Scientific and Technological Research, Pathum Thani 12120, Thailand; wiriyaporn@tistr.or.th; 5 Department of Biochemistry, Faculty of Medical Science, Naresuan University, Phitsanulok 65000, Thailand; anyapata@nu.ac.th (A.A.); kittiphumm66@nu.ac.th (K.M.); 6 Center for Computational Sciences, University of Tsukuba, 1-1-1 Tennodai, Tsukuba 305-8577, Ibaraki, Japan; shigeta@ccs.tsukuba.ac.jp (Y.S.); kowith@ccs.tsukuba.ac.jp (K.H.); 7 Biomedical Engineering Institute, Chiang Mai University, Chiang Mai 50200, Thailand; sarawut.kumphune@cmu.ac.th; 8 Biomedical Engineering and Innovation Research Center, Chiang Mai University, Mueang Chiang Mai District, Chiang Mai 50200, Thailand

**Keywords:** lotus seed, rat aorta, vasorelaxation, nitric oxide, hypotensive effect

## Abstract

**Background/Objectives:** Enhancing endothelial nitric oxide (NO) bioavailability through natural products may provide a promising strategy for the prevention and management of hypertension. This study investigated the phytochemical composition of ethanolic lotus (*Nelumbo nucifera*) seed extract (LSE), its vasorelaxant mechanisms, effects on endothelial NO production, and antihypertensive activity. **Methods:** LSE was characterized via LC-ESI-QTOF-MS using accurate mass data and fragmentation patterns. Vasorelaxant effects were evaluated in isolated rat aortas, and the underlying mechanisms were explored using pharmacological inhibitors. NO production was assessed in human endothelial EA.hy926 cells. Hypotensive activity was examined in normotensive rats following intravenous administration of LSE (10, 30, and 100 mg/kg). Molecular docking was performed to analyze interactions between LSE bioactive compounds and endothelial nitric oxide synthase (eNOS). **Results:** LC-ESI-QTOF-MS analysis identified 114 compounds, including primary and secondary metabolites. LSE induced vasorelaxation in endothelium-intact aortas, which was reduced by endothelium removal (*p* < 0.001) and by L-NAME (*p* < 0.001). LSE also inhibited receptor-operated, Ca^2+^ channel-mediated vasoconstriction (*p* < 0.05). In vivo, LSE decreased blood pressure in a dose-dependent manner. In EA.hy926 cells, LSE (750 and 1000 µg/mL) increased NO production, an effect attenuated by L-NAME. Molecular docking showed that LSE alkaloids, including nelumborine, nelumboferine, neferine, and isoliensinine had strong affinities for binding with eNOS at the tetrahydrobiopterin (BH4) binding site. Nelumborine exhibited the highest affinity, suggesting its potential as an eNOS modulator. **Conclusions:** LSE promotes vasorelaxation through the stimulation of endothelium-derived NO release and Ca^2+^ influx inhibition, contributing to blood pressure reduction. These findings support LSE as a potential natural antihypertensive supplement.

## 1. Introduction

Hypertension, a key risk factor for cardiovascular events such as myocardial infarction and stroke, is associated with endothelial dysfunction, increased vascular contraction, and arterial remodeling [1,2]. The vascular endothelium regulates blood pressure by releasing mediators such as endothelium-derived relaxing factors (EDRFs) and contracting factors (EDCFs) [3]. Nitric oxide (NO), synthesized by endothelial nitric oxide synthase (eNOS), is the most critical EDRF, and its reduced bioavailability is central to hypertension-related endothelial dysfunction [4]. Plant-derived substances are increasingly studied for their ability to activate eNOS, enhancing NO release, vasorelaxation, and cardiovascular protection [5].

Lotus (*Nelumbo nucifera*), an aquatic perennial flowering plant from the Nymphaeaceae family, is also known by various names such as water lily, sacred lotus, and Kamala. This plant thrives in diverse regions across Asia. All parts of the lotus, including its rhizome, stems, leaves, flowers, and seeds, are valued for their therapeutic potential [6,7,8]. Lotus seeds are a crucial part of the plant, valued for their rich nutritional and bioactive components including phenolic compounds, flavonoids, and alkaloids, which contribute to a range of biological effects [6,7,9]. Thus, the rapid elucidation of the chemical structures of the active components is essential for advancing promising candidates to drug development or food supplement phases. Lotus seeds are extensively used in both culinary and medicinal applications across tropical regions such as Thailand, Vietnam, India, and China [6]. Previous studies have demonstrated that ethanolic lotus seed extract (LSE) exhibits antioxidant [10], anti-inflammatory [11], anti-cancer [12], anti-fertility [13], and anti-proliferative [14] effects. Recently, our in vivo study demonstrated that oral LSE lowered blood pressure and improved cardiovascular morphology in N^ω^-nitro-L-arginine methyl ester (L-NAME)-induced hypertensive rats, alongside the upregulation of aortic eNOS and increases in circulating nitrate/nitrite, with the concurrent attenuation of oxidative stress markers [15]. However, key questions remain unclear, including whether LSE directly modulates vascular reactivity and enhances endothelial NO production at the cellular level, and how its acute hemodynamic effects manifest in normotensive animals. In addition, a comprehensive chemical characterization of ethanolic LSE and an in silico interrogation of putative eNOS-interacting constituents are needed to connect chemistry with biology. Therefore, the present study aimed to (i) systematically characterize both known and novel chemical constituents of LSE using accurate mass data and fragmentation patterns through reverse-phase high-performance liquid chromatography coupled with mass spectrometry (LC-ESI-QTOF-MS); (ii) investigate the vasorelaxant effect and its underlying mechanisms, including the binding pattern and susceptibility of potential active components of LSE to eNOS using molecular docking; (iii) evaluate LSE cytotoxicity and its effect on endothelial NO production in human endothelial cells (EA.hy926); (iv) examine the acute hypotensive effect of LSE in normotensive rats.

## 2. Results

### 2.1. Phytochemical Screening of LSE by LC-ESI-QTOF/MS

**Figure 1:**
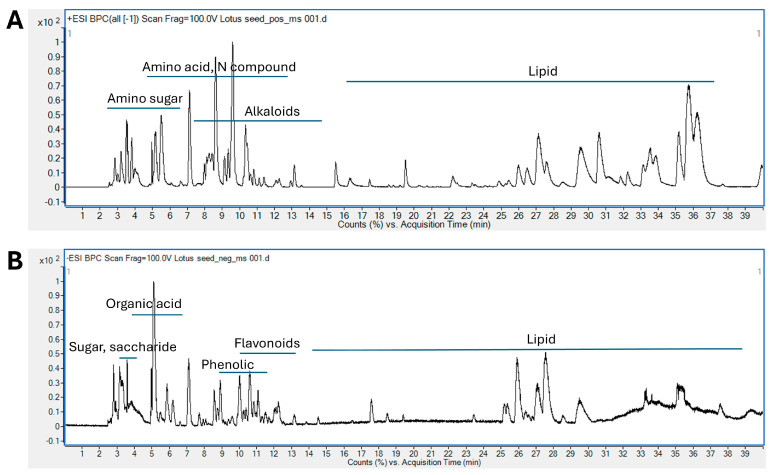
LC-ESI-QTOF-MS elution profiles base peak chromatogram of LSE at concentration 20 mg/mL. (**A**) positive mode, (**B**) negative mode.

The chemical profile of LSE is illustrated in Figure 1. Mass spectrometry provides both qualitative (mass accuracy) and quantitative (concentration or amount found) data by converting analytes into ions. In LC-MS analysis, ion abundances are presented in a total ion chromatogram (TIC), which shows peak intensities against retention time (RT). Each point on the TIC corresponds to a mass spectrum, plotting ion abundances against the mass-to-charge ratio (*m*/*z*). When interpreting mass spectrometry data, it is crucial to consider the ionization mode. Electrospray ionization (ESI) is a soft ionization technique where, in positive mode, adducts such as [M+NH_4_]^+^, [M+Na]^+^ or [M+K]^+^ can appear, while in negative mode, adducts like [M+HCOO]^−^ and [M+Cl]^−^ are commonly found. Fragmentation patterns reveal gaps between fragment ions, which indicate neutral losses (e.g., −18 Da for H_2_O, −44 Da for CO_2_) or specific moieties (e.g., −162 Da for hexose or caffeic acid). The elution order in reverse-phase chromatography is influenced by the LogP (partition coefficient). The nitrogen rule helps determine whether an analyte contains an odd or even number of nitrogen atoms [16]. Comparing the results with existing databases and literature enhances the interpretation and validation of the data.

**Figure 2:**
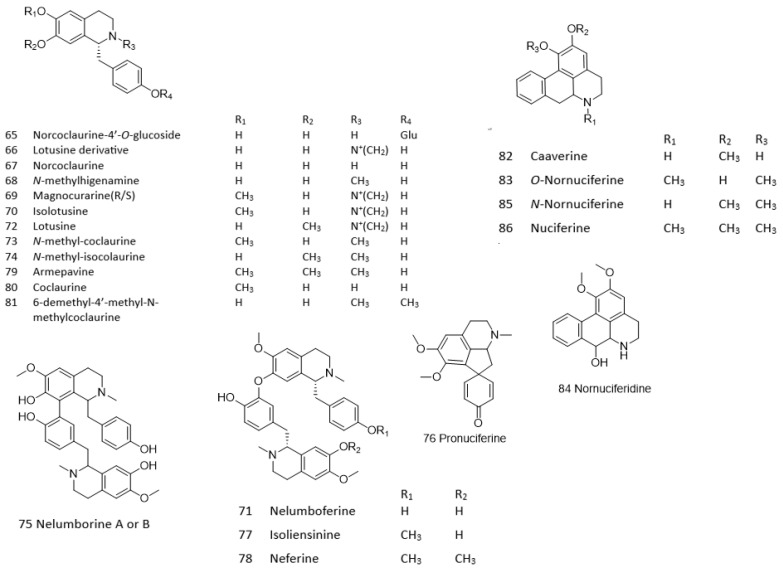
Structure proposed of alkaloids found in lotus seed extract.

The phytochemical compounds in lotus seeds were tentatively identified using high-resolution LC-ESI-QTOF-MS. Table 1 lists the accurate mass and fragmentation data for various primary and secondary metabolites. A total of 114 compounds were identified in lotus seeds, comprising amino sugars (1–8), organic acids (9–14), saccharides (15–22), amino acids and nitrogenous compounds (23–41), phenolics (42–50), flavonoids (51–64), alkaloids (65–86), and fatty acids (87–114). The study further explored both bioactive secondary metabolites and primary metabolites. Several distinct classes of flavonoids were identified, including *C*-glycosides and *O*-glycosides, along with alkaloids such as benzylisoquinoline, bisbenzylisoquinoline, aporphine, and proaporphine (Figure 2). These secondary metabolites have received significant attention due to their pharmacological effects.

### 2.2. LSE-Induced Endothelium-Dependent and Endothelium-Independent Vasorelaxant Effects

**Figure 3:**
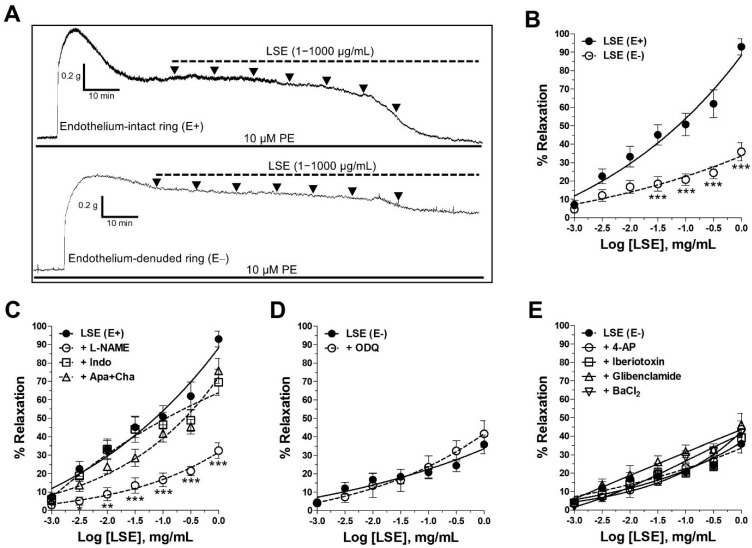
Effects of LSE on the rat thoracic aorta, endothelial signaling pathway, the sGC pathway, and K^+^ channels. (**A**) Representative traces of the vasorelaxant effect of LSE in endothelium-intact (E+) and -denuded (E−) rings precontracted with 10 µM PE. The black arrowheads along the tracing indicate the time points at which LSE was cumulatively added at increasing concentrations (1–1000 µg/mL). (**B**) Concentration–response curves (CRCs) of LSE in endothelium-intact (E+) and -denuded (E−) rings. (**C**) CRCs of LSE after incubation with or without 100 µM L–NAME, 10 µM indomethacin, or 0.1 µM apamin + 0.1 µM charybdotoxin in endothelium-intact (E+) or with (**D**) 10 µM ODQ or (**E**) various K^+^ channel inhibitors: 1 mM 4–AP (K_V_ blocker), 0.1 µM iberiotoxin (K_Ca_ blocker), 10 µM glibenclamide (K_ATP_ blocker), and 1 mM BaCl_2_ (K_IR_ blocker) in endothelium-denuded (E−) rings. Values are means ± SEM, of n individual arteries (n = 5–8). * *p* < 0.05, ** *p* < 0.01, *** *p* < 0.001 vs. LSE (E+).

LSE induced a concentration-dependent vasorelaxation in endothelium-intact (E+, EC_50_ = 0.34 ± 0.10 mg/mL and E_max_ = 93.9 ± 4.4%) and endothelium-denuded rings (E−, EC_50_ > 1000 mg/mL and E_max_ = 35.9 ± 5.0%) (Figure 3A,B). The removal of the endothelium significantly blunted the relaxant effect of LSE (*p* < 0.001, Figure 3B), as confirmed by the 2.6-fold decrease in the E_max_ values. After washout, 80 mM high-potassium (K^+^) solution re-elicited contractions did not significantly differ from pre-LSE responses. 

### 2.3. Mechanism of Vasorelaxant Action of LSE via NO Pathway and Receptor-Operated Ca^2+^ Channels (ROCCs) Inhibition

In endothelium-intact rings, the inhibition of NOS by L-NAME markedly reduced LSE-induced relaxation (EC_50_ > 1 mg/mL and E_max_ = 32.5 ± 4.1%, *p* < 0.001, +L-NAME vs. LSE (E+) alone, Figure 3C). In contrast, the blockade of cyclooxygenase (COX) by indomethacin or inhibition of endothelium-derived hyperpolarizing factors (EDHFs) using apamin plus charybdotoxin modestly reduced the maximal response (E_max_ = 69.5 ± 7.1%, *p* < 0.05 and 75.7 ± 6.8%, n.s., respectively) without a rightward shift in EC_50_ (0.34 ± 0.04 and 0.40 ± 0.07 mg/mL, respectively).

In endothelium-denuded rings, the blockers including 1H-[1,2,4]oxadiazolo [4,3-a]quinoxaline-1-one (ODQ), 4-aminopyridine (4–AP), iberiotoxin, glibenclamide and barium chloride (BaCl_2_), which inhibited soluble guanylyl cyclase (sGC), voltage-gated K^+^ channels (K_V_), large conductance calcium (Ca^2+^)-activated K^+^ channels (K_Ca_), ATP-sensitive K^+^ channels (K_ATP_) and inward-rectifying K^+^ channels (K_IR_), respectively, did not alter the vasorelaxation induced by LSE (EC_50_ > 1 mg/mL and E_max_ = 41.6 ± 7.1%, 43.7 ± 4.5%, 39.2 ± 4.3%, 46.2 ± 6.3% and 41.7 ± 2.2%, respectively, Figure 3D,E).

**Figure 4:**
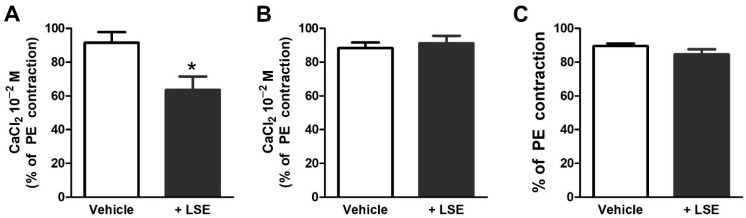
Effects of LSE on Ca^2+^ channels. (**A**,**B**) Effect of LSE on endothelium-denuded aortic ring contraction induced by extracellular Ca^2+^ influx via (**A**) receptor operated Ca^2+^ channels (ROCCs), which were activated by 10 µM PE or via (**B**) voltage-operated Ca^2+^ channels (VOCCs), which were activated by 80 mM K^+^ solution. The sarcoplasmic reticulum (SR) Ca^2+^ depletion was performed before activation of ROCCs and VOCCs followed by addition of CaCl_2_ solution to evoke contraction in Ca^2+^-free Krebs solution. The bar graphs represent the percentage of the 10 mM CaCl_2_-induced contraction in Ca^2+^-free Krebs solution in the presence of either vehicle or LSE normalized with 10 µM PE -induced contraction in normal Krebs solution without vehicle or LSE. (**C**) Effect of LSE on endothelium-denuded aortic ring contraction induced by Ca^2+^ release from SR activated by 10 µM PE. The SR Ca^2+^ loading was performed before activation of Ca^2+^ release from SR in Ca^2+^-free Krebs solution. The bar graphs represent the percentage of the contraction induced by 10 µM PE in Ca^2+^-free Krebs solution in the presence of either vehicle or LSE normalized with 10 µM PE-induced contraction without vehicle or LSE. Values are means ± SEM n individual arteries (n = 5–8). * *p* < 0.05 vs. vehicle.

LSE decreased the contraction induced by extracellular Ca^2+^ influx in endothelium-denuded aortic rings exposed to phenylephrine (PE) (opening of ROCCs, Figure 4A), but not the high (80 mM) K^+^ solution (opening of voltage-operated Ca^2+^ channels (VOCCs), Figure 4B). It did not affect the contraction induced by the intracellular Ca^2+^ release from the sarcoplasmic reticulum (SR, Figure 4C).

### 2.4. Cytotoxicity and NO Production in EA.hy926 Human Endothelial Cells Treated with LSE

**Figure 5:**
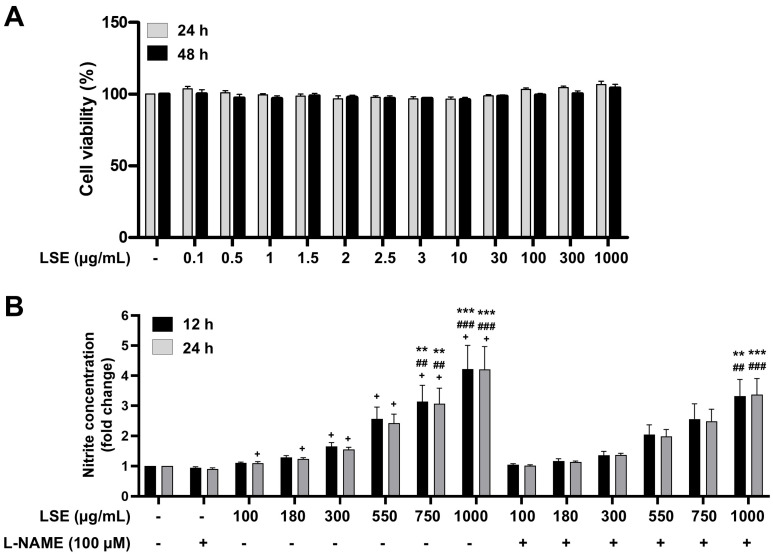
Effect of LSE on the viability and NO production of EA.hy926 human endothelial cells. (**A**) The percentage of cell viability of EA.hy926 human endothelial cells incubated with various concentrations of LSE for 24 and 48 h. (**B**) Nitrite levels in cell culture media of EA.hy926 human endothelial cells treated with various concentrations of LSE for 12 and 24 h in the absence or presence of 100 µM L-NAME. Values are means ± SEM of at least 3–4 independent experiments, each experiment performed in triplicate. ** *p* < 0.01, *** *p* < 0.001 vs. untreated cells; ^##^ *p* < 0.01, ^###^ *p* < 0.001 vs. the cells treated with only L-NAME; ^+^ *p* < 0.05 vs. the presence of L-NAME and treatment with LSE at the same concentration and duration.

The incubation of EA.hy926 human endothelial cells in cell culture media supplemented with LSE (0.1–1000 µg/mL) for 24 and 48 h did not affect cell viability (Figure 5A). In addition, LSE (750 and 1000 µg/mL) significantly enhanced NO production in EA.hy926 human endothelial cells compared to both the untreated group and the cells treated with L-NAME alone. Notably, even in the presence of 100 µM L-NAME, treatment with 1000 µg/mL LSE still significantly enhanced NO production compared to the untreated group and the cells treated with L-NAME alone (Figure 5B).

### 2.5. LSE-Induced Acute Hypotensive Effect

**Figure 6:**
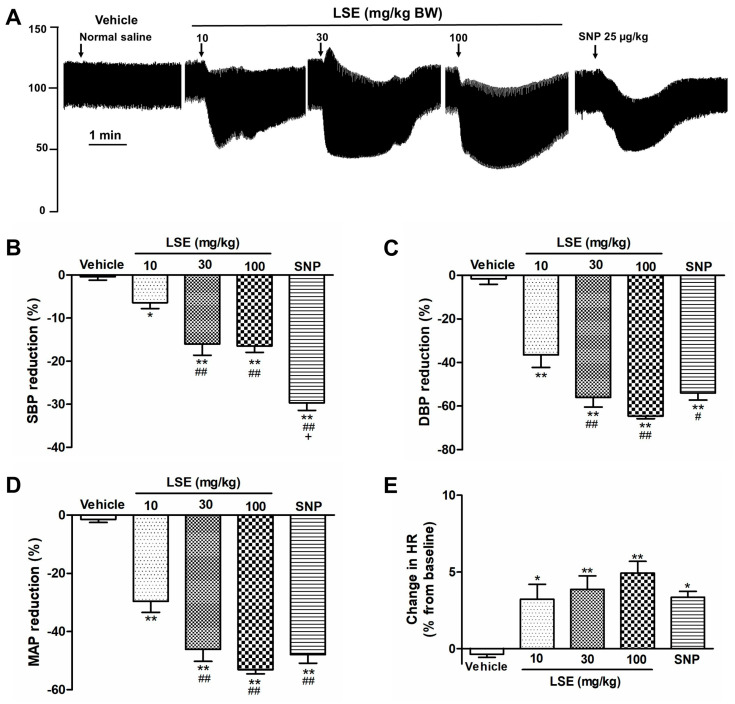
Acute effect of LSE intravenous injection on blood pressure and heart rate in normotensive anesthetized rats. (**A**) Original traces showing arterial pressure responses to intravenous injections of vehicle (normal saline), LSE (10, 30, 100 mg/kg BW), and SNP (25 µg/kg BW), with arrows indicating administration times. Bars represent the percentage reductions in (**B**) systolic, (**C**) diastolic, (**D**) mean arterial pressure, and (**E**) the percentage change in heart rate from baseline. Values are mean ± SEM, (*n* = 8); * *p* < 0.05, ** *p* < 0.01 vs. vehicle, ^#^ *p* < 0.05, ^##^ *p* < 0.01 vs. LSE 10 mg/kg, ^+^ *p* < 0.01 vs. LSE 100 mg/kg.

Baseline cardiovascular values without treatment were systolic blood pressure (SBP) = 126.6 ± 4.2 mmHg, diastolic blood pressure (DBP) = 86.8 ± 2.1 mmHg, mean arterial pressure (MAP) = 100.1 ± 2.1 mmHg and heart rate (HR) = 453.6 ± 6.9 beats per minute. Intravenous injection of the vehicle (normal saline) had no effect (Figure 6), while LSE (10, 30, and 100 mg/kg) decreased SBP, DBP, and MAP (*p* < 0.05, *p* < 0.01 vs. vehicle, Figure 6B–D). The percentage increases in HR were observed after the intravenous injections of LSE (10, 30, and 100 mg⁄kg) and sodium nitroprusside (SNP, Figure 6E).

### 2.6. Molecular Docking

**Figure 7:**
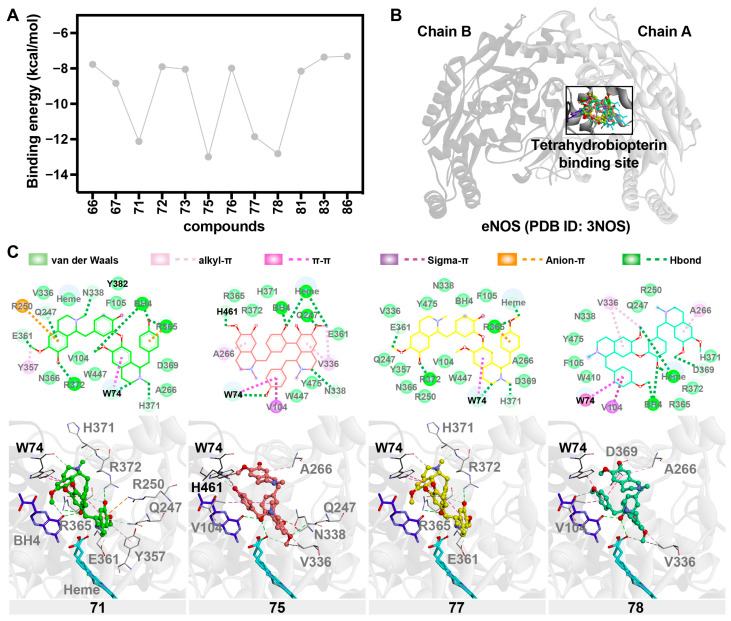
Molecular docking analysis of compounds with eNOS. (**A**) The binding energy profile of compounds 66–86 docked with eNOS illustrates the variability in binding affinity (kcal/mol). (**B**) Structure of eNOS (PDB ID: 3NOS) depicting Chain A and Chain B, and the location of the tetrahydrobiopterin (BH4) binding site where compounds interact. (**C**) Detailed 2D and 3D interaction analysis of representative docked compounds 71, 75, 77, and 78 with eNOS residues.

The molecular docking analysis evaluated the binding interactions between bioactive compounds from LSE and eNOS (PDB ID: 3NOS). The study focused on the tetrahydrobiopterin (BH4) binding site, a crucial region for eNOS function, as BH4 is an essential cofactor for eNOS. Among the screened bisbenzylisoquinoline compounds, 71 (nelumboferine), 75 (nelumborine A or B), 77 (isoliensinine), and 78 (neferine) exhibited strong binding affinities, with docking scores ranging from −11.858 to −12.999 kcal/mol, suggesting their potential as potent eNOS bioactive modulators (Figure 7A,B).

All four compounds formed key interactions within the BH4 binding pocket. Hydrogen bonding was observed with Q247, R365, and N338, stabilizing the ligands within the site. Additionally, van der Waals interactions with residues V336, N366, W447, and heme contributed to the binding stability. Notably, π–π stacking interactions with W74 were consistent across all four compounds, further enhancing their binding affinities.

Among these, compound 75 (nelumborine A or B with two *N*-methylcoclaurine units linked by C–C bond) exhibited the strongest binding affinity (−12.999 kcal/mol). This can be attributed to its unique sigma–π interaction with H461 and strong alkyl–π stacking with W74, which may contribute to its superior stability in the binding pocket. Compound 71 (nelumboferine with two *N*-methylcoclaurine units linked by a C–O bond) displayed an additional anion–π interaction with R250, further strengthening its electrostatic stabilization. Compound 77 (isoliensinine with dimer of *N*-methylcoclaurine and 4′-*O*-methyl-*N*-methylcoclaurine via a C–O linkage) formed a notable π–π stacking interaction with W74, while compound 78 (neferine with armepavine and 4′-*O*-methyl-*N*-methylcoclaurine, connected by a C–O bond) exhibited multiple hydrogen bonds with BH4, R365, and H371, as well as alkyl-π and π–π stacking interactions with W74 and V104. Thus, the molecular docking results suggest that compounds 71, 75, 77, and 78 from LSE could act as potential bioactive ligands for eNOS, particularly by targeting the BH4 binding site. Their strong binding affinities and diverse interaction profiles support their potential effectiveness (Figure 7C). Nevertheless, as each compound’s interaction with eNOS may result in either activation or inhibition, the overall effect is likely to promote NO release and vasorelaxation.

## 3. Discussion

The major findings of this study demonstrate that ethanolic lotus seed extract (LSE) induces vasorelaxation via both endothelium-dependent and -independent mechanisms. These effects are mediated by the stimulation of endothelium-derived NO release and the inhibition of Ca^2+^ influx through ROCCs, cumulating in an acute hypotensive response in normotensive anesthetized rats. The pharmacological benefits of LSE are further supported by its ability to enhance NO production in EA.hy926 human endothelial cells, along with its lack of cytotoxicity. Molecular docking also revealed strong binding affinities between the LSE active compounds and eNOS, confirming the central role of eNOS/NO pathway in the vascular action of LSE.

Vascular tone plays a pivotal role in blood pressure regulation and is governed by a complex interplay between vasodilators and vasoconstrictors released by the vascular endothelium [17,18,19]. In this study, LSE induced concentration-dependent vasorelaxation in both endothelium-intact and endothelium-denuded aortic rings, with a more pronounced effect observed in the presence of the endothelium. After washout, the high K^+^ (80 mM) contraction recovered to pre-LSE levels, indicating that LSE-induced relaxations were reversible and not attributable to tissue deterioration. The endothelium-dependent relaxation was blocked by an eNOS inhibitor, whereas COX inhibition produced a modest reduction in E_max_ without altering EC_50_, and EDHF blockade did not significantly affect either parameter.

In endothelium-denuded rings, the sGC inhibitor, ODQ, did not modify the relaxation evoked by LSE, suggesting that LSE did not directly activate vascular smooth muscle sGC. Nevertheless, the role of sGC as the downstream molecular target of NO is crucial for LSE-induced vasorelaxation. Measuring cGMP levels in vascular smooth muscle could further confirm the role of the eNOS/NO/sGC pathway involved in the mechanisms of action of LSE. Furthermore, LSE could possibly influence endothelial calcium dynamics and ROS, which can indirectly affect eNOS activity, often through complex crosstalk [20]. These areas could be a focus for future study to fully clarify LSE’s mechanism of action. Taken together, our results indicate that the NO pathway is primarily responsible for the endothelial component of LSE-induced vasorelaxation. This finding was further supported by the increased NO production observed in EA.hy926 human endothelial cells treated with LSE, an effect that was attenuated by an eNOS inhibitor, thereby confirming the involvement of the eNOS/NO pathway in LSE-induced vasorelaxation. These results are concordant with a recent in vivo study showing that oral LSE lowered blood pressure in L-NAME-induced hypertensive rats while upregulating aortic eNOS and increasing plasma nitrate/nitrite, alongside reductions in oxidative stress markers, collectively consistent with improved NO bioavailability [15].

Our data also revealed that the vasorelaxant effect of LSE is partly mediated through endothelium-independent mechanisms, which may involve interference with the ion channels on vascular smooth muscle cells (VSMCs) membranes, such as K^+^ and Ca^2+^ channels [17,21]. VSMCs express at least four different types of K^+^ channels, including K_v_, K_ATP_, K_Ca_, and K_IR_ channels whose activation leads to membrane hyperpolarization, subsequently inhibiting Ca^2+^ influx through L-type Ca^2+^ channels and promoting VSMCs relaxation [17,22]. However, our findings indicate that LSE does not affect these K^+^ channels. Therefore, we examined whether LSE interferes with extracellular Ca^2+^ influxes through VOCCs and ROCCs as well as intracellular Ca^2+^ release from SR through IP_3_ receptors [21,22]. Our results demonstrated that LSE selectively attenuated Ca^2+^-induced vascular contraction following PE stimulation, indicating that its vasorelaxant effect involves the blockade of ROCC-mediated Ca^2+^ entry, while it had no effect on intracellular Ca^2+^ release from SR. Therefore, the primary molecular target underlying the endothelium-independent vasorelaxant action of LSE is the inhibition of ROCCs.

Various bioactive compounds in LSE may contribute to its vascular effects, particularly through endothelium-dependent vasorelaxation mediated by NO. In the present study, the chemical constituents of LSE were comprehensively characterized using liquid chromatography–electrospray ionization–quadrupole time-of-flight mass spectrometry (LC-ESI-QTOF-MS). This advanced analytical technique relies on high-resolution mass measurements, mass accuracy, and a detailed analysis of adduct formation, neutral losses, and fragmentation patterns. By integrating these components with database matching and fragmentation patterns, LC-ESI-QTOF-MS serves as a powerful tool for the systematic identification of both known and novel compounds in plant and natural product research. Employing LC-ESI-QTOF-MS, we identified 114 compounds in LSE, providing a comprehensive profile of its chemical constituents, including amino sugars, organic acids, saccharides, amino acids and nitrogenous compounds, phenolics, flavonoids, alkaloids, and fatty acids. Notably, the presence of diverse flavonoids, such as catechin, kaempferol, apigenin, and rutin, and alkaloids, including benzylisoquinoline (e.g., lotusine, isolotusine, norcoclaurine, *N*-methyl-coclaurine, 6-demethyl-4′-methyl-*N*-methylcoclaurine), bisbenzylisoquinoline (e.g., nelumboferine, isoliensinine, neferine, nelumborines A or B), and aporphine (e.g., nuciferine, *O*-nornuciferine, *N*-nornuciferine, caaverine, pronuciferine), aligns with previous findings that highlight the pharmacological significance of these secondary metabolites [6,23,24]. Twelve alkaloid compounds were subsequently selected for molecular docking screening based on their pharmacological relevance in elucidating the mechanisms underlying LSE-induced, eNOS/NO-dependent vasorelaxation. Among these, four compounds (nelumboferine, nelumborine A or B, isoliensinine, and neferine) demonstrated promising potential for direct interaction with eNOS. A molecular docking study suggested that these compounds exhibit strong affinities with eNOS by binding to the BH4 cofactor site, with binding affinities reaching −12.999 kcal/mol. These molecular interactions, including hydrogen bonding with Q247, R365, and N338, as well as π–π stacking with W74, are consistent with findings from similar studies [25,26,27]. This is in line with the observed vasorelaxant properties of LSE, which are attributed to enhanced endothelial NO production. We also found that E361 and D369 act as crucial residues similar to those observed in the previous study [28]. Therefore, these data support the hypothesis that the endothelium-dependent vasorelaxant effect of LSE is mediated, at least in part, by increased NO bioavailability through direct interactions between its active constituents and eNOS. While molecular dynamics (MD) simulations could add further insight, our docking results are supported by LSE-enhanced NO release, L-NAME–sensitive vasorelaxation, and hypotension in vivo. The stabilization of the BH4 site promotes eNOS coupling and NO generation, whereas its loss causes uncoupling [29,30]. Thus, LSE alkaloids likely stimulate rather than inhibit eNOS activity.

The ex vivo data obtained from isolated thoracic aorta suggest that LSE may exert a hypotensive effect in vivo. Consistent with this, our in vivo results demonstrated that the intravenous infusion of LSE in rats produced a dose-dependent decrease in SBP, DBP, and MAP. The LSE’s effect was greater on DBP than SBP, suggesting that its primary mechanism is a decrease in total peripheral resistance, which is largely regulated by resistance arteries. While our study used conduit arteries (thoracic aorta), the findings are relevant because both conduit and resistance arteries share similar vasodilation pathways. Though the dominant vasoactive factor may vary (e.g., NO in conduit arteries vs. EDHF in resistance arteries), the underlying signaling is similar [31]. Therefore, our results provide valuable insight into LSE’s vascular mechanism, while future studies could directly investigate its effect on resistance arteries. In contrast to a reduction in blood pressure, an increase in heart rate was observed following treatment with either LSE or sodium nitroprusside, a potent vasodilator. This response is likely due to reflex tachycardia, a compensatory physiological mechanism commonly triggered by vasodilatory agents to maintain blood pressure homeostasis, particularly when the vasodilator is administered acutely via intravenous injection, as in this study. However, in our previous work using L-NAME-induced hypertensive rats, we found that the oral administration of LSE reduced both blood pressure and heart rate compared with hypertensive controls [15]. These findings suggest that reflex tachycardia may not be a prominent side effect under pathological conditions, and, therefore, may not limit the potential clinical use of LSE.

The hypotensive effect observed in the present study may be attributed to the synergistic actions of multiple compounds present in LSE. Among the identified alkaloid constituents, only neferine has been previously reported to exhibit antihypertensive and vasorelaxant effects via the eNOS/NO/sGC pathway and Ca^2+^ antagonism [32,33]. Further investigation is warranted to explore other active compounds and their potential synergistic interactions. Collectively, these findings suggest that the systemic hypotensive effects of LSE are primarily mediated through vasodilation, supporting its potential as a natural antihypertensive supplement.

## 4. Materials and Methods

### 4.1. Plant Extract Preparation

Lotus seeds were collected from Nakhon Sawan, Thailand, and authenticated with a voucher specimen (BK No. 082574). Dry powder (15 kg) was soaked in 95% ethanol (75 L) and sonicated for 30 min. After 1 h, the mixture was filtered, and the extraction process repeated twice. The combined filtrates were evaporated at 47 °C to yield 3.82 ± 0.56% crude ethanolic extract, which was stored at −20 °C until used. High-performance liquid chromatography (HPLC) analyses were performed to monitor the chemical profile and verify the quality of the extract in accordance with previously published methods [34].

### 4.2. Phytochemical Analysis Using LC-ESI-QTOF-MS

The chemical constituents of LSE were analyzed following the methodology outlined by Chansriniyom et al. (2021) [35]. The separation process was conducted using an Agilent 1260 Infinity Series HPLC system (Agilent, Waldbronn, Germany) equipped with a Luna C18(2) column (4.6 mm × 150 mm, 5 μm). Chemical characterization was performed using a (+/−) ESI-QTOF mass spectrometer (Agilent 6540, Singapore). Phytochemical compounds were identified based on peak retention time, accurate mass data, and fragmentation patterns, which were compared to entries in the Human Metabolome Database (HMDB) and the METLIN Metabolomics Database and Library (Agilent Technologies, Santa Clara, CA, USA). For compounds lacking database references, identification was inferred from their fragmentation patterns.

### 4.3. Animals

Adult male Wistar rats (8–12 weeks old) were purchased from Nomura Siam International Co., Ltd. (Bangkok, Thailand) and housed under standard conditions (22 ± 1 °C, 12:12 h light/dark cycle) with free access to food and water at the Center for Animal Research, Naresuan University. All protocols were approved by the Naresuan University Animal Care and Use Committee (Approval number: NU-AE650709 and NU-AE630301).

### 4.4. Vasorelaxant Effects of LSE on Isolated Rat Thoracic Aorta

*Tissue preparation and vascular protocols.* The rats were anesthetized with sodium thiopental (60 mg/kg, intraperitoneal injection), and then the thoracic aorta was isolated and placed in cold Krebs solution. This Krebs solution consisted of the following concentrations in mM: NaCl 122, KCl 5, *N*-[2-Hydroxyethyl] piperazine-*N*′-[2-ethane-sulfonic acid] (HEPES) 10, KH_2_PO_4_ 0.5, NaH_2_PO_4_ 0.5, MgCl_2_ 1, CaCl_2_ 1.8, and glucose 11. The solution was adjusted to pH 7.4 with 1 M NaOH. Afterward, the aorta was cleaned, cut into approximately 2 mm lengths, and suspended in Krebs solution at 37 °C and aerated for isometric tension recording in organ chambers, as previously described [36]. The functional integrity of the aortic rings was verified by applying an 80 mM high K^+^ solution at the beginning and end of each experiment. The presence of functional endothelium was confirmed by a relaxation of ≥80% to 10 µM acetylcholine (ACh) in vessels precontracted with 10 µM PE. Endothelium-denuded rings exhibited no relaxation to ACh. Subsequently, the rings were constricted with 10 µM PE, and cumulative concentration-response curves to LSE were obtained by adding 1, 3, 10, 30, 100, 300, and 1000 µg/mL sequentially to both endothelium-intact and endothelium-denuded rings.

*Mechanism of LSE-induced vasorelaxation*. The contribution of endothelium-dependent pathways, the sGC pathway, vascular smooth muscle K^+^ channels, and intracellular/extracellular Ca^2+^ fluxes were assessed. To explore the role of eNOS, COX, and EDHF, endothelium-intact rings were pretreated with 100 µM L-NAME (an eNOS inhibitor), 10 µM indomethacin (a COX inhibitor), or 0.1 µM apamin plus 0.1 µM charybdotoxin (small- and large-conductance Ca^2+^-activated K^+^ channel blockers), respectively, for 30 min [36] before contraction with 10 µM PE and exposure to cumulative concentrations of LSE. The role of sGC in vasorelaxant activity was assessed by incubating endothelium-denuded aortic rings with 10 µM ODQ (a selective inhibitor of NO-sensitive sGC) for 30 min before contraction with 10 µM PE and exposure to cumulative concentrations of LSE. The role of vascular smooth muscle K^+^ channels in vasorelaxant activity was assessed by incubating endothelium-denuded aortic rings with 1 mM 4-AP (K_V_ blocker), 10 µM glibenclamide (K_ATP_ blocker), 0.1 µM iberiotoxin (K_Ca_ blocker) or 1 mM BaCl_2_ (K_IR_ blocker) for 30 min [36] before contraction with 10 µM PE and exposure to cumulative concentrations of LSE. To assess the involvement of extracellular Ca^2+^ influx in LSE-induced vasorelaxation, endothelium-denuded rings were incubated in Ca^2+^-free Krebs solution containing 2 mM ethylene glycol-bis(2-aminoethylether)-*N*,*N*,*N*’,*N*’-tetraacetic acid (EGTA) for 40 min. Then 10 µM PE was added to deplete intracellular Ca^2+^ store from SR. After 4 washes with Ca^2+^-free Krebs solution every 10 min, rings were incubated with vehicle (distilled water) or LSE at the EC_50_ (340 µg/mL) for 10 min in Ca^2+^-free Krebs solution with 10 µM PE to open the ROCCs or with Ca^2+^-free 80 mM K^+^ solution to open the VOCCs. Then, cumulative concentrations of CaCl_2_ (0.01–10 mM) were added to evoke a contractile response. The contractions evoked by CaCl_2_ were normalized as percentage to 10 µM PE-induced contraction in the normal Krebs solution in the same aortic ring and % maximum contractions to 10 mM CaCl_2_ were compared between conditions with or without LSE [36]. To investigate the effect of LSE on intracellular Ca^2+^ release from the SR via IP_3_ receptors, endothelium-denuded rings were precontracted with 80 mM K^+^ solution for 5 min to stimulate the initial Ca^2+^ loading into the SR Ca^2+^ stores. Then, baths were replaced with Ca^2+^-free Krebs solution for 15 min and 10 µM PE was added to release Ca^2+^ from SR, thereby eliciting a transient contraction. Then the same protocol was repeated after incubation for 10 min with vehicle (distilled water) or LSE at the EC_50_ (340 µg/mL) before adding 10 µM PE [36].

### 4.5. Cytotoxicity and NO Production Effects of LSE on EA.hy926 Human Endothelial Cells

*Cytotoxic effects of LSE on the viability of EA.hy926 human endothelial cells.* The human endothelial cell line (EA.hy926; ATCC: CRL-2922) were cultured in a 96-well plate at a density of 10,000 cells/well in Dulbecco’s Modified Eagle Medium (DMEM) supplemented with 10% (*v*/*v*) fetal bovine serum (Gibco, Thermo Fisher Scientific, Waltham, MA, USA), 100 units/mL penicillin and 100 µg/mL streptomycin (Gibco) and incubated in a cell culture incubator at 37 °C, 5% CO_2_ for 24 h. Subsequently, the culture medium was removed and replaced with medium containing LSE at concentrations ranging from 0.1–1000 µg/mL. The cells were then further incubated for an additional 24 and 48 h under the same conditions. Effect of LSE on the viability of EA.hy926 cells was assessed using the PrestoBlue^TM^ cell viability reagent (Invitrogen, Thermo Fisher Scientific, Waltham, MA, USA), with absorbance measured at 570 nm as the experimental wavelength and 600 nm as the normalization wavelength using a Varioskan^TM^ LUX multimode microplate reader (Thermo Fisher Scientific, Waltham, MA, USA).

*Effects of LSE on NO production*. Human endothelial cells EA.hy926 were cultured in a 96-well plate at a density of 10,000 cells/well and incubated at 37 °C, 5% CO_2_ for 24 h. Afterwards, the cells were washed twice with 1X PBS and then incubated in serum-free media without phenol red for 1 h. Following this, the cells were incubated in serum-free media without phenol red supplemented with LSE at concentrations ranging from 0.1–1000 µg/mL at 37 °C, 5% CO_2_ for 12 and 24 h. Subsequently, the amount of NO was measured by indirect detection of nitrite (NO_2_^−^) produced from the spontaneous oxidation of NO using Griess assay. This was achieved by mixing 100 µL of cell culture medium with an equal volume of Griess reagent (0.1% *N*-(1-naphthyl) ethylenediamine in phosphoric acid and 1% sulfanilamide), thoroughly mixing the components, and allowing the mixture to incubate in darkness at room temperature for 15 min. Absorbance was then measured at a wavelength of 540 nm using a Varioskan^TM^ LUX multimode microplate reader (Thermo Scientific). The nitrite levels were expressed as a relative fold change to the value of control samples (only DMEM), which was set at 1.

### 4.6. Acute Hypotensive Effect of LSE

To investigate the acute hypotensive effect of LSE compared to SNP, rats were anesthetized with a 1% isoflurane-oxygen mixture, a concentration well below the higher levels (≥3%) reported to significantly influence blood pressure [37]. Then SBP, DBP, MAP, and HR were measured following cannulation of the left carotid artery and connection of the catheter to a pressure recording system, as previously described [15]. After a 15 min stabilization period, all parameters were recorded before (baseline) and during intravenous infusion via the right femoral vein of 1 mL/kg vehicle (normal saline), LSE (10, 30, or 100 mg/kg), or SNP (25 μg/kg). The doses of LSE were selected based on preliminary studies to ensure a dose–response relationship of LSE and capture its full pharmacological spectrum from a submaximal effect to a maximal response. Changes in blood pressure were expressed as percentages of baseline values recorded immediately before drug injection.

### 4.7. Molecular Docking

Molecular docking studies were performed to investigate the binding modes and affinities of selected compounds towards eNOS (PDB ID: 3NOS [38]). Twelve out of twenty-two alkaloids found in LSE were selected for molecular docking studies due to their wide ranges of therapeutic effects. They were categorized into four structural classes: **(i) Benzylisoquinolines:** this class includes lotusine derivatives such as compound 66 and lotusine (72), both characterized by a quaternary amine at the nitrogen center. Norcoclaurine (67) features a primary amine (NH) at the nitrogen center, while *N*-methylcoclaurine (73) and 6-demethyl-4′-methyl-*N*-methylcoclaurine (81) possess nitrogen centers conjugated with a methyl group. **(ii) Bisbenzylisoquinolines**: This class comprises compounds such as nelumboferine (71) and nelumborines A or B (75), both of which consist of two *N*-methylcoclaurine units connected via a C–O bond (71) or a C–C bond (75). Additional representatives include isoliensinine (77) and neferine (78). **(iii) Aporphines**: This class includes *O*-nornuciferine (83) and nuciferine (86). **(iv) Proaporphines**: This class includes pronuciferine (76). Prior to docking, both the protein and ligand structures were prepared using ADCP tools [39]. Hydrogen atoms were added to both the protein and ligands, considering protonation states at pH 7.4. Subsequently, the structures were converted to the PDBQT file format, which is compatible with AutoDock Vina [40].

Docking simulations were carried out using AutoDock Vina 1.2.3 [40], as previously described [41,42,43]. For each compound, ten independent docking runs were performed, generating 40 poses per run, with an exhaustiveness setting of 32 to ensure thorough sampling of the conformational space. The top-ranked pose, based on the Vina scoring function, was selected from each of the ten runs. The pose with the lowest binding energy among the ten iterations was then chosen to represent the compound’s binding affinity and used for generating the binding energy line graph. To further analyze the interaction details, the binding poses of selected, high-affinity compounds were visualized and analyzed using Discovery Studio Visualizer [44]. This allowed for the identification of key interacting residues and the characterization of the types of interactions, such as hydrogen bonds, hydrophobic interactions, and electrostatic interactions, contributing to the binding affinity of the compounds.

### 4.8. Statistical Analysis

All data are presented as mean ± SEM (n = animals or independent cultures). Vasorelaxation (%) by LSE was calculated relative to 10 µM PE-induced contraction. EC_50_ and E_max_ values were determined using GraphPad Prism (V5.0). Concentrationresponse relationships were compared by two-way analysis of variance (ANOVA) for repeated measures. Multiple comparisons were analyzed using one-way ANOVA followed by Tukey’s test. Comparison between two values was assessed by unpaired Student’s *t*-test. *p* < 0.05 was considered statistically significant.

## 5. Conclusions

This study provides a multi-faceted analysis of the hypotensive mechanisms of LSE. We first identified a complex profile of 114 compounds and demonstrated that LSE-induced vasorelaxation occurs via endothelium-dependent NO release and the inhibition of Ca^2+^ influx through ROCCs. Molecular docking further identified several compounds, with nelumborine showing the highest affinity for the eNOS BH4 domain. These actions contribute to the observed dose-dependent acute hypotensive effect of LSE. In conclusion, our findings provide a strong pharmacological basis for LSE as a potential natural antihypertensive supplement. Future research should focus on validating its long-term efficacy and safety in hypertensive animal models, such as spontaneously hypertensive rats or two-kidney one-clip hypertensive rats, and subsequently in clinical trials. Furthermore, isolating specific bioactive compounds, particularly nelumborine, is necessary for future drug development.

## Figures and Tables

**Table 1:** MS data (+/−ESI) and tentative identification of compounds found in LSE.

No	RT(min)	*m*/*z*	Adduct	MS/MS	Tentative Identification	Formula	Error (ppm)
	Amino sugars						
1	2.811	264.1086	[M−H]^−^	174.0740,**102**.0543	*N*-fructosyl-GABA	C_10_H_19_NO_7_	−2.74
		266.1239	[M+H]^+^	248.1135,182.0816,116.0709, **104**.0706,98.0602	*N*-fructosyl-GABA	C_10_H_19_NO_7_	−1.77
2	2.988	250.0935	[M−H]^−^	130.0490,**88**.0393	*N*-fructosyl alanine	C_9_H_17_NO_7_	−1.1
		252.1085	[M+H]^+^	234.0979,216.0868,188.0919, 170.0818,**90**.0552	*N*-fructosyl alanine	C_9_H_17_NO_7_	−2.86
3	3.554	280.1392	[M+H]^+^	216.1238,**118**.0866,72.0810	*N*-fructosyl valine	C_11_H_21_NO_7_	−0.43
4	4.971	290.0898	[M−H]^−^	200.0538,**128**.0332	*N*-Fructosyl pyroglutamate	C_11_H_17_NO_8_	−2.96
5	5.054	344.1343	[M+H]^+^	326.1246,308.1133,280.1185, **182**.0805	*N*-fructosyl tyrosine	C_15_H_21_NO_8_	−0.89
6	5.249	294.1551	[M+H]^+^	276.1451,230.1394,**132**.1020, 86.0966	*N*-fructosyl isoleucine	C_12_H_23_NO_7_	−1.26
7	7.112	328.1396	[M+H]^+^	310.1294,292.1179,264.1242, **166**.0883,120.0813	*N*-fructosyl phenylalanine	C_15_H_21_NO_7_	−1.59
8	8.362	365.136	[M−H]^−^	**203**.0800,101.0236	*N*-Fructosyl-tryptophan	C_17_H_22_N_2_O_7_	−1.58
	8.408	367.1504	[M+H]^+^	349.1395,229.0980,188.0717, 146.0606,118.0650,85.0283	*N*-Fructosyl-tryptophan	C_17_H_22_N_2_O_7_	−1.15
	Sugar						
9	3.047	665.2082	[M−H]^−^	503.1555,383.1115,179.0520, 89.0225	Tetraglucoside	C_24_H_42_O_21_	9.59
10	3.102	181.0723	[M−H]^−^	89.0228,59.0137	*D*-manitol	C_6_H_14_O_6_	−2.97
11	3.144	549.1579	[M+HCOO]^−^	503.1623,341.1019,323.0906, 179.0535,89.0231	Triglucose (Maltotriose)	C_18_H_32_O_16_	−1.08
12	3.22	537.167	[M−H]^−^	503.1521,341.1032,195.0472, 129.0185,75.0080	Gluconic acid glycoside	C_18_H_34_O_18_	−2.35
13	3.295	683.2262	[2M−H]^−^	341.1028,179.0633,89.0230	Sucrose	C_12_H_22_O_11_	−1.54
14	3.493	179.0568	[M−H]^−^	89.0230,59.0135	Fructose	C_6_H_12_O_6_	−3.84
	Organic acids						
15	3.572	133.0146	[M−H]^−^	115.0021,71.0138	Malic acid	C_4_H_6_O_5_	0.35
16	5.01	191.0202	[M−H]^−^	111.0075,87.0079,57.0346	Citric acid	C_6_H_8_O_7_	−2.48
17	5.868	117.0236	[M−H]^−^	73.0292	Succinic acid	C_4_H_6_O_4_	−0.58
18	6.042	115.004	[M−H]^−^	71.0135	Maleic acid	C_4_H_4_O_4_	−2.76
19	6.09	133.0509	[M−H]^−^	71.0134	4,5-Dihydroxypentanoic acid	C_5_H_10_O_4_	−2.01
20	6.21	161.0461	[M−H]^−^	99.0441,57.0345	3-Hydroxy-2-methylglutaric acid	C_6_H_10_O_5_	−1.57
21	9.467	117.0557	[M−H]^−^	73.0281,71.049	5-Hydroxypentanoic acid	C_5_H_10_O_3_	0.15
22	10.269	175.0616	[M−H]^−^	157.0489,115.0326	2-Isopropylmalic acid	C_7_H_12_O_5_	−2.3
	Amino acids & N-compounds						
23	2.528	156.0772	[M+H]^+^	110.0713	Histidine	C_6_H_9_N_3_O_2_	−2.86
24	2.531	175.1197	[M+H]^+^	158.0927,130.0975,116.0710	Arginine	C_6_H_14_N_4_O_2_	−4.27
25	2.762	207.2075	[2M−H]^+^	104.1073	2-Amino-3-methyl-1-butanol	C_5_H_13_NO	−3.84
26	2.812	174.0772	[M−H]−	145.0613,102.0546,59.0129	*N*-Carboxyethyl-gramma-aminobutyric acid	C_7_H_13_NO_4_	−0.11
27	3.228	138.0549	[M+H]^+^	94.0662,92.0496	*p*-Aminobenzoic acid	C_7_H_7_NO_2_	0.4
28	3.258	116.0709	[M+H]^+^	70.0654	Proline	C_5_H_9_NO_2_	
29	3.514	118.086	[M+H]^+^	72.0810	Valine	C_5_H_11_NO_2_	2.16
30	3.62	182.0816	[M+H]^+^	165.0639,147.0441,136.0759, 107.0490,91.0543	Tyrosine	C_9_H_11_NO_3_	−2.36
31	3.815	132.1019	[M+H]^+^	86.0967	Beta leucine	C_6_H_13_NO_2_	0.04
32	5.077	130.05	[M+H]^+^	84.0448	Pyroglutamic acid	C_5_H_7_NO_3_	−1.0
	5.119	128.0351	[M−H]^−^	71.0129,52.0188	Pyroglutamic acid	C_5_H_7_NO_3_	0.13
33	5.138	268.1046	[M+H]^+^	136.0623,57.0337	Adenosine	C_9_H_17_NO_8_	−7.11
34	5.173	132.1021	[M+H]^+^	86.0968,56.0496	Leucine	C_6_H_13_NO_2_	0.04
35	5.513	132.1016	[M+H]^+^	86.0968	Isoleucine	C_6_H_13_NO_2_	2.31
	5.501	130.0875	[M−H]^−^	71.0138,59.0396	Isoeucine	C_6_H_13_NO_2_	3.82
36	6.089	160.0763	[M+H]^+^	132.0810,115.0544,86.0964, 72.0444,61.0283	Indoleacetaldehyde	C_10_H_9_NO	−3.81
37	7.131	166.0863	[M+H]^+^	120.0810,77.0389	Phenylalanine	C_9_H_11_NO_2_	−0.27
	7.142	164.0722	[M−H]^−^	147.0430,103.0547,77.0388	Phenylalanine	C_9_H_11_NO_2_	2.14
38	7.687	220.1184	[M+H]^+^	202.1077,184.0968,90.0551	Pantothenic acid	C_9_H_17_NO_5_	−2.05
39	8.583	203.083	[M−H]^−^	116.0496,74.0244	Tryptophan	C_11_H_12_N_2_O_2_	2.03
	8.593	205.0976	[M+H]^+^	188.0712,146.0606,118.0653, 74.0231	Tryptophan	C_11_H_12_N_2_O_2_	−2.17
40	11.092	210.0776	[M−H]^−^	179.0343,124.0390,94.0288	3-Methoxy-DL-tyrosine	C_10_H_13_NO_4_	−1.99
41	12.53	289.0834	[M−H]^−^	173.0688,132.0275,88.0394	L-N-(*1H*-Indol-3-yl acetylaspartic acid)	C_14_H_14_N_2_O_5_	−1.4
	Phenolic compounds						
42	4.978	180.1024	[M+NH_4_]^+^	163.0757,145.0652,117.0700, 91.0543	4-Methylcinnamic acid	C_10_H_10_O_2_	−2.75
43	6.662	194.1183	[M+NH_4_]^+^	177.0788,91.0534	Cinnamyl acetate	C_11_H_12_O_2_	−3.84
44	8.766	371.0929	[M+HCOO]^−^	163.0382,119.0496	6-*O*-*p*-Coumaroyl-*D*-glucose	C_15_H_18_O_8_	−1.97
45	9.088	153.0194	[M−H]^−^	109.0285,81.0331	Protocatechuic acid	C_7_H_6_O_4_	−0.44
46	9.595	421.0909	[M+Cl]^−^	385.1084,265.0660,223.0586, 179.0635	1-*O*-Sinapoylglucose	C_17_H_22_O_10_	−0.48
47	10.986	137.0245	[M−H]^−^	93.0334,65.0387	3-Hydroxybenzoic acid	C_7_H_6_O_3_	−0.6
48	11.265	179.035	[M−H]^−^	135.0432,107.0491	Caffeic acid	C_9_H_8_O_4_	−0.1
49	11.511	165.0194	[M−H]^−^	121.0279,77.0392	1,4-benzendicarboxylic acid	C_8_H_6_O_4_	−0.41
50	13.164	163.0382	[M−H]^−^	119.0486	*p*-coumaric acid	C_9_H_8_O_3_	11.46
	Flavonoids						
51	9.601	577.1349	[M−H]^−^	451.0956,425.0812,407.0716, 289.0675,245.0412,125.0230	Procyanidin B2	C_30_H_26_O_12_	0.43
52	9.924	593.1522	[M−H]^−^	503.1130,473.1004,383.0708, 353.0591,289.0672,139.0036	Apigenin-6,8-di-*C*-glycopyranoside (Vicenin-2)	C_27_H_30_O_15_	−1.7
53	10.03	289.0722	[M−H]^−^	245.0781,203.0684,125.0229, 109.0283	Catechin	C_15_H_14_O_6_	−1.52
54	10.615	563.1411	[M−H]^−^	503.1100,473.1010,443.0908, 383.0712,353.0618,325.0660, 297.0707	Apigenin 6-*C*-glucosyl-8-*C*-arabinoside (Schaftoside)	C_26_H_28_O_14_	−0.84
	10.621	565.1565	[M+H]^+^	529.1347,481.1134,427.1032, 379.0830,325.0712	Apigenin 6-*C*-glucoside 8-*C*-arabinoside (Schaftoside)	C_26_H_28_O_14_	−2.33
55	10.768	449.1087	[M−H]^−^	357.0542,329.0636,287.0511, 259.0581,125.0229	Eriodictyol 7-*O*-glucoside	C_21_H_22_O_11_	0.52
56	10.847	563.142	[M−H]^−^	503.1000,443.0886,353.0612, 125.0224	Apigenin 6-*C*-arabinoside 8-*C*-glucoside (Isoschaftoside)	C_26_H_28_O_14_	−0.84
	10.851	565.1563	[M+H]^+^	427.1033,379.0814,325.0699	Apigenin 6-*C*-arabinoside 8-*C*-glucoside (Isoschaftoside)	C_26_H_28_O_14_	−2.33
57	10.963	447.0939	[M−H]^−^	357.0556,327.0451,285.0361	Luteolin 6-*C*-glucoside (Isorientin)	C_21_H_20_O_11_	−1.38
		449.108	[M+H]^+^	413.0869,329.0653,243.0290	Luteolin 6-*C*-glucoside (Isorientin)	C_21_H_20_O_11_	−0.36
58	11.517	577.1559	[M−H]^−^	487.1164,457.1079,383.0706, 353.0605,325.0670,289.0655, 179.0522,89.0231	Apigenin 6-*C*-glucosyl-8-*C*-rhamnoside	C_27_H_30_O_14_	0.66
59	11.712	609.1469	[M−H]^−^	300.0222,301.0296,151.0014	Rutin	C_27_H_30_O_16_	−1.3
	11.71	611.1619	[M+H]^+^	465.1035,303.0505129.0546, 85.0283	Rutin	C_27_H_30_O_16_	−2.03
60	11.943	593.1511	[M−H]^−^	285.0361,151.0018	Kaempferol 3-*O*-rutinoside	C_27_H_30_O_15_	0.16
	11.958	595.1661	[M+H]^+^	541.2672,449.1087,287.0551, 249.1143,192.1003	Kaempferol 3-*O*-rutinoside	C_27_H_30_O_15_	−0.59
61	12.029	431.098	[M−H]^−^	311.0510,283.0564,223.0922	Apigenin 8-*C*-glucoside	C_21_H_20_O_10_	0.86
	12.03	433.1136	[M+H]^+^	313.0711,283.0605	Apigenin 8-*C*-glucoside (Vitexin)	C_21_H_20_O_10_	−1.56
62	12.369	463.0877	[M−H]^−^	301.0287,151.0006	Quercetin 3′-*O*-glucoside	C_21_H_20_O_12_	1.08
	12.431	465.1031	[M+H]^+^	303.0502,145.0496,85.0284	Quercetin 3′-*O*-glucoside	C_21_H_20_O_12_	−0.75
63	12.642	625.1765	[M+H]^+^	479.1185,317.0658,129.0541	Isorhamnetin-3-*O*-*b*-*D*- rutinoside (Narcissoside)	C_28_H_32_O_16_	−0.3
	12.576	623.1618	[M−H]^−^	315.0456,271.0187,151.0355	Isorhamnetin-3-*O*-*b*-*D*- rutinoside (Narcissoside)	C_28_H_32_O_16_	−0.07
64	13.463	447.0863	[M−H]^−^	285.0369,151.0001,137.0245, 59.0131	Kaempferol 3-*O*-glucoside	C_21_H_20_O_11_	15.62
	Alkaloids						
65	6.614	432.1666	[M−H]^−^	270.1102,162.0540,96.9579	Norcoclaurine-4′-*O*-glucoside	C_12_H_27_NO_8_	−0.49
	6.614	434.1816	[M+H]^+^	272.1282,255.1025,194.1170, 161.0603,107.0494	Norcoclaurine-4′-*O*-glucoside	C_12_H_27_NO_8_	−1.51
66	7.753	300.1603	M^+^	255.1006,107.0493,58.0653	Lotusine derivative	C_18_H_21_NO_3_	−2.93
67	7.976	272.1289	[M+H]^+^	255.1023,209.0968,161.0699, 107.0495	Norcoclaurine	C_16_H_17_NO_3_	−2.87
	7.961	270.1151	[M−H]^−^	249.1187,162.0546,135.0425, 114.0439,59.0139	Norcoclaurine	C_16_H_17_NO_3_	−5.68
68	8.082	284.1296	[M−H]^−^	219.0605,176.0699,107.0494	*N*-methylhigenamine	C_17_H_19_NO_3_	−1.35
	8.111	286.144	[M+H]^+^	255.1017,107.0493,58.0653	*N*-methylhigenamine	C_17_H19NO_3_	−0.8
69	8.235	314.1745	M^+^	269.1181,237.0914,175.0756, 107.0493,58.0654	Magnocurarine	C_19_H_24_NO_3_^+^	1.81
70	8.409	314.1756	M^+^	269.1180,237.0917,209.0945, 175.0757,145.0652,107.0494, 58.0653	Isolotusine	C_19_H_24_NO_3_^+^	−1.69
	8.56	312.1624	[M−H]^−^	297.1327,239.0680,119.0490	Isolotusine	C_19_H_24_NO_3_	−6.03
71	8.525	299.1526	[M+2H]^+^	269.1181,237.0917,192.1025, 107.0495,58.0653	Nelumboferine	C_36_H_40_N_2_O_6_	−3.29
		597.2969	[M+H]^+^		Nelumboferine	C_36_H_40_N_2_O_6_	−3.83
72	8.573	312.1609	[M−H]^−^	297.1327,239.0680,163.0383, 119.0490,93.0341,59.0127	Lotusine	C_19_H_23_NO_3_	−1.23
	8.625	314.1755	M^+^	269.1177,237.0911,209.0966, 175.0759,107.0493,58.0654	Lotusine	C_19_H_23_NO_3_	−1.37
73	9.135	300.1598	[M+H]^+^	269.1178,237.0915,175.0758, 137.0599,107.0495,58.0653	*N*-methyl-coclaurine	C_18_H_21_NO_3_	−1.27
74	9.346	300.1597	[M+H]^+^	269.1181,237.0918,209.0968, 175.0759,137.0594,107.0494, 77.0387,58.0652	*N*-methyl-isococlaurine	C_18_H_21_NO_3_	−0.93
75	9.431	299.1526	[M+2H]^+^	269.1185,192.1023,137.0596, 107.0491,58.0647	Nelumborine A or B	C_36_H_40_N_2_O_6_	−3.29
		597.2982	[M+H]^+^	597.2982,405.1902,300.1586, 269.1168,192.1023,107.0500	Nelumborine A or B	C_36_H_40_N_2_O_6_	−3.83
76	9.533	312.1601	[M+H]^+^	283.1346,269.1185,206.1173, 149.0226,107.0492,58.0652	Pronuciferine	C_19_H_21_NO_3_	2.18
77	9.6	306.1602	[M+2H]^+^	192.1025,158.0734,121.0653, 91.0546	Isoliensinine	C_37_H_42_N_2_O_6_	−2.32
		611.3131	[M+H]^+^	537.2224,475.2244,312.1597, 192.1027,121.0631,58.0652	Isoliensinine	C_37_H_42_N_2_O_6_	−2.51
78	10.275	313.1679	[M+2H]^+^		Neferine	C_38_H_44_N_2_O_6_	−1.7
		625.3295	[M+H]^+^	594.2868,582.2857,489.2401, 206.1178	Neferine	C_38_H_44_N_2_O_6_	−3.66
79	10.371	314.1752	[M+H]^+^	283.1338,252.1163,107.0496, 77.0389,58.0652	Armepavine	C_19_H_23_NO_3_	−0.41
80	10.815	286.1444	[M+H]^+^	269.1179,175.0754,143.0495, 107.0495	Coclaurine	C_17_H_19_NO_3_	−2.2
81	10.839	300.1601	[M+H]^+^	269.1171,237.0912,192.1021, 163.0737,143.0490,107.0494, 77.0386,58.0652	6-demethyl-4′-methyl-*N*-methylcoclaurine	C_18_H_21_NO_3_	−1.53
82	11.117	268.1339	[M+H]^+^	251.1074,219.0808,191.0864, 149.0236	Caaverine	C_17_H_17_NO_2_	−2.59
83	11.394	282.1489	[M+H]^+^	251.1490,219.0808,191.0859, 149.0234	*O*-Nornuciferine	C_18_H_19_NO_2_	−0.16
84	11.913	298.1447	[M+H]^+^	251.1076,219.0806,191.0863, 121.0664,74.0957	Nornuciferidine	C_18_H_19_NO_3_	−3.12
85	12.925	282.1497	[M+H]^+^	265.1228,250.0992,234.1041, 219.0815,149.0234,74.0965	*N*-Nornuciferine	C_18_H_19_NO_2_	−2.99
86	13.135	296.165	[M+H]^+^	265.1229,250.0990,234.1040, 219.0802	Nuciferine	C_19_H_21_NO_2_	−1.67
	Lipid compounds						
87	17.585	327.2184	[M−H]^−^	211.1315,171.1008	9,12,13-Trihydroxy- 10(*E*),15(*Z*)-octadeca dienoic acid	C_18_H_32_O_5_	−2.15
88	18.493	329.2336	[M−H]^−^	211.1317,171.1004,99.0807	9,10,13-Trihydroxy-11-octadecenoic acid	C_18_H_34_O_5_	−0.77
89	19.193	298.2746	[M+H]^+^	280.2632,250.2519	Palmitoleoyl Ethanolamine	C_18_H_35_NO_2_	−1.82
90	19.512	318.3011	[M+H]^+^	282.2787,219.1741,60.0443	Phytosphingosine	C_18_H_39_NO_3_	−2.61
91	22.476	366.3375	M^+^	307.2627,129.0546,81.0699	Linoleoylcholine	C_23_H_44_NO_2_	−0.81
92	23.33	342.3372	M^+^	283.2632,95.0843	*N,N,N*-trimethyl-sphingosine	C_21_H_44_NO_2_	0.01
93	23.448	711.3362	[M+Cl]^−^	595.2801,397.1294,277.2134	3′-*O*-Linolenoylglyceryl 6-*O*-galactopyranosyl-galactopyranoside	C_33_H_56_O_14_	0.29
94	25.432	478.2938	M^+^	337.2737,263.2370,184.0728, 81.0699	1-Linoleoyl-2-hydroxy-*sn*-glycero-3-phosphoethanolamine	C_23_H_44_NO_7_P	−2.06
	25.945	476.2768	[M−H]^−^	279.2280,196.0340,140.0087, 78.9580	LysoPE(0:0/18:2(*9Z*,*12Z*))	C_23_H_44_NO_7_P	3.07
95	26.421	554.3006	[M+Cl]^−^	504.3001,433.2276,279.2274, 78.9569	1-Linoleoyl-glycero-3-phosphocholine	C_26_H_50_NO_7_P	2.33
	26.489	520.3407	M^+^	337.2734,258.1098,184.0734, 86.0964	1-Linoleoyl-glycero-3-phosphocholine	C_26_H_51_NO_7_P	−0.74
96	26.594	689.3499	[M+Cl]^−^	653.3615,397.1276,279.2263, 255.2270,179.0541	1-palmitoyl-2-azeloyl-*sn*-glycero-3-phospho-(1′-*sn*-glycerol)	C_31_H_59_O_12_P	−8.83
97	27.112	554.3009	[M+Cl]^−^	504.2997,279.2281,168.0394, 78.9575	1-(*2E,4E*-octadecadienoyl)-*sn*-glycero-3-phosphocholine	C_26_H_50_NO_7_P	2.33
	27.156	520.3405	M^+^	443.2518,337.2736,258.1096, 184.0735,104.1068	1-(*2E,4E*-octadecadienoyl)-*sn*-glycero-3-phosphocholine	C_26_H_51_NO_7_P	−0.36
98	27.565	452.2767	[M−H]^−^	255.2281,196.0345,140.0090, 78.9579	2-Palmitoyl-*sn*-glycero-3-phosphoethanolamine	C_21_H_44_NO_7_P	3.46
	27.613	454.2934	M^+^	436.2824,393.2403,313.2741, 155.0098,62.0600	2-Palmitoyl-*sn*-glycero-3-phosphoethanolamine	C_21_H_44_NO_7_P	−1.29
99	28.532	496.3408	[M+H]^+^	184.0735,104.1068	1-tetradecyl-2-acetyl-*sn*- glycero-3-phosphocholine	C_24_H_50_NO_7_P	−2.08
100	28.571	478.2928	[M−H]^−^	281.2428,152.9916,78.9582	1-Oleoyl-2-hydroxy-*sn*-glycero-3-PE	C_23_H_46_NO_7_P	2.33
	28.629	480.3091	[M+H]^+^	419.2544,339.2893,184.0736	1-Oleoyl-2-hydroxy-*sn*-glycero-3-PE	C_23_H_46_NO_7_P	−1.32
101	28.64	551.2976	[M+Cl]^−^	515.3118,279.2281,152.9920	3′-*O*-Linoleoyglyceryl-6-*O*-galactopyranoside	C_27_H_48_O_9_	2.96
102	28.701	615.3032	[M+Cl]^−^	579.3273,341.1049,255.2275, 179.0542	1-palmitoyl-2-azeloyl-*sn*-glycero-3-phosphate	C_28_H_53_O_10_P	6.23
103	29.58	496.3405	[M+H]^+^	419.2551,184.0736,104.1069	2-Palmitoyl-*sn*-glycero-3-phosphocholine	C_24_H_50_NO_7_P	−1.48
	29.725	530.3014	[M+Cl]^−^	480.3013,409.2263,339.3201, 255.2289,168.0405,78.9566	2-Palmitoyl-*sn*-glycero-3-phosphocholine	C_24_H_50_NO_7_P	0.93
104	31.798	366.3001	[M+NH_4_]^+^	261.2201,86.0599	2-hydroxy-6-pentadecyl benzoic acid	C_22_H_36_O_3_	0.47
105	31.823	358.2518	[M+Cl]^−^		Linoleoyl ethanolamide	C_20_H_37_NO_2_	0.09
	31.856	324.2896	[M+H]^+^	245.2240,62.0598	Linoleoyl ethanolamide	C_20_H_37_NO_2_	0.33
106	32.266	403.2329	[M+H]^+^	365.1930,259.1541,185.0808, 129.0180,61.0282	*5S*-HETE di-endoperoxide	C_20_H_34_O_8_	−0.63
107	33.635	389.2463	[M+Cl]^−^	279.2268,152.9958	2,3-dihydroxypropyl (*9Z,12Z*)-octadeca-9,12-dienoate	C_21_H_38_O_4_	0.29
108	33.635	699.5029	[M−H]^−^	437.2563,279.2274,181.0258	Octadecanoyl-(*9Z,12Z*-octadecadienoyl)-*sn*-glycero-3-phosphate	C_39_H_73_O_8_P	−8.39
109	33.649	280.2631	[M+H]^+^	263.237,245.2265,198.1855, 149.0229,95.0833,81.0699, 69.0698	Linoleamide	C_18_H_33_NO	1.4
110	35.189	256.2641	[M+H]^+^	116.1070,102.0913,88.0757	Palmitamide	C_16_H_33_NO	−2.38
111	35.242	699.4941	[M−H]^−^	437.2562,279.2280,255.2289, 134.8921	1-(9*Z*,12*Z*-octadecadienoyl)-2-octadecanoyl-glycero-3-phosphate	C_39_H_73_O_8_P	4.19
112	35.734	282.2805	[M+H]^+^	256.2634,97.1010,83.0856 69.0699,55.0541	Oleamide	C_18_H_35_NO	−4.81
113	36.239	282.2812	[M+H]^+^	83.0855,69.0698,55.0542	Elaidamide	C_18_H_35_NO	−7.29
114	39.9	284.2955	[M+H]^+^	130.1220,102.0914,88.0754	Steramide	C_18_H_37_NO	−2.49

## Data Availability

The raw data supporting the conclusions of this article will be made available by the authors upon request.
